# Irisin Protects Musculoskeletal Homeostasis via a Mitochondrial Quality Control Mechanism

**DOI:** 10.3390/ijms251810116

**Published:** 2024-09-20

**Authors:** Chong Zhao, Yonghao Wu, Shuaiqi Zhu, Haiying Liu, Shuai Xu

**Affiliations:** Department of Spinal Surgery, Peking University People’s Hospital, Peking University, Beijing 100871, China

**Keywords:** irisin, mitochondrial quality control, musculoskeletal health, myokine, skeletal muscle, osteoporosis

## Abstract

Irisin, a myokine derived from fibronectin type III domain-containing 5 (FNDC5), is increasingly recognized for its protective role in musculoskeletal health through the modulation of mitochondrial quality control. This review synthesizes the current understanding of irisin’s impact on mitochondrial biogenesis, dynamics, and autophagy in skeletal muscle, elucidating its capacity to bolster muscle strength, endurance, and resilience against oxidative-stress-induced muscle atrophy. The multifunctional nature of irisin extends to bone metabolism, where it promotes osteoblast proliferation and differentiation, offering a potential intervention for osteoporosis and other musculoskeletal disorders. Mitochondrial quality control is vital for cellular metabolism, particularly in energy-demanding tissues. Irisin’s influence on this process is highlighted, suggesting its integral role in maintaining cellular homeostasis. The review also touches upon the regulatory mechanisms of irisin secretion, predominantly induced by exercise, and its systemic effects as an endocrine factor. While the therapeutic potential of irisin is promising, the need for standardized measurement techniques and further elucidation of its mechanisms in humans is acknowledged. The collective findings underscore the burgeoning interest in irisin as a keystone in musculoskeletal health and a candidate for future therapeutic strategies.

## 1. Introduction

Mitochondrial quality control (MQC) is a key factor in maintaining muscle health, and its dysregulation is associated with a variety of diseases, including muscle atrophy, diabetes, cardiovascular diseases, and others [1,2,3]. Skeletal muscle, as the largest metabolic organ in the human body, has a direct impact on the overall metabolic balance and physical performance. Mitochondria play an essential role in skeletal muscle, not only participating in energy metabolism but also involved in various biological processes such as cell signaling, cell death, and more [4,5]. MQC in skeletal muscle cells occurs via a complex and delicate regulatory network, involving biosynthesis, a dynamic balance, autophagy, and responses to nutritional and environmental changes [6,7]. In recent years, a large number of studies have focused on the mechanisms of MQC in skeletal muscle and its role in diseases. A deep understanding of these mechanisms is of great significance for the prevention and treatment of diseases related to mitochondrial dysfunction.

Myokines are a class of proteins or peptides secreted by skeletal muscle cells that can regulate the signal transduction of skeletal muscle itself and the metabolism of peripheral tissues and organs through autocrine, paracrine, or endocrine mechanisms [8]. Since the identification of interleukin (IL)-6 as the first myokine in 2003, research on myokines has rapidly increased, and the number of identified myokines has also grown, such as IL-6, myostatin, growth and differentiation factor 11 (GDF11), fibroblast growth factor 21 (FGF21), and irisin [9]. Irisin is an exercise-induced myokine derived from the cleavage of fibronectin type III domain-containing 5 (FNDC5) and has been extensively studied in recent years for its role in skeletal muscle health. Studies have shown that irisin, by binding to the αV/β5 integrin on the membranes of osteocytes and adipocytes [10], exerts positive effects through various mechanisms, including MQC [11,12,13]. During exercise, the release of irisin promotes mitochondrial biogenesis and function, enhancing the energy metabolic capacity of muscle cells, thereby improving muscle strength and endurance [14,15]. Additionally, irisin has been found to protect skeletal muscle cells from oxidative stress damage by regulating mitochondrial autophagy, which is crucial for maintaining muscle health and preventing muscle atrophy [16,17].

In summary, the muscle-protective effects of irisin have been confirmed by in vivo and in vitro experiments, and its specific mechanisms of action are also a hot topic of current research. Mitochondria, as the center of energy metabolism, also execute intracellular signal cascades through the production of reactive oxygen species (ROS), fatty acid β-oxidation, amino acid metabolism, pyridine synthesis, phospholipid modification, calcium regulation, and other pathways. Their complex functions and the threats they face determine the high dynamics of mitochondria. Through MQC, which includes fission, fusion, and autophagy, they promptly remove damaged mitochondria and form new mitochondria to adapt to the metabolic needs of the cell [18]. This article reviews the key role of MQC in skeletal muscle cells and the effect mediated by irisin in this process.

## 2. Mitochondrial Quality Control in Skeletal Muscle Stability

**Figure 1:**
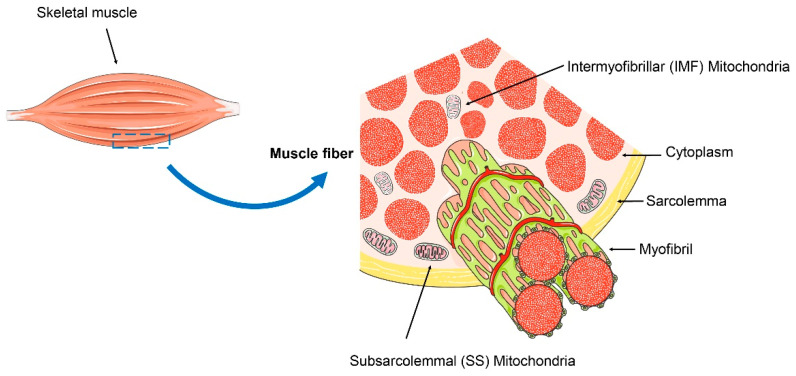
Subsarcolemmal (SS) mitochondria and intermyofibrillar (IMF) mitochondria in the skeletal muscle. SS mitochondria are typically located beneath the sarcolemma, while IMF mitochondria are situated in the spaces between myofibrils.

Mitochondrial homeostasis in skeletal muscle cells is essential for maintaining the normal physiological functions. Skeletal muscle, serving as the body’s primary organ for movement and heat production, endures substantial metabolic demands. Mature skeletal muscle fibers accumulate hundreds to thousands of nuclei derived from resident muscle stem cells (MuSCs) through a process of cellular fusion [19]. These fibers are replete with mitochondria, which are capable of modulating their metabolic capacity in response to physiological stimuli. The skeletal muscle has even evolved a specialized mitochondrial system to match its high energy expenditure, with mitochondria classified into subsarcolemmal (SS) mitochondria and intermyofibrillar (IMF) mitochondria. SS mitochondria, typically located beneath the sarcolemma, constitute about 20% of the total mitochondrial population in skeletal muscle, while IMF mitochondria, situated in the spaces between myofibrils, account for the remaining 80% [20,21] (Figure 1). A hallmark of skeletal muscle aging is mitochondrial dysfunction, characterized by morphological abnormalities (such as swelling, increased branching, and structural damage like ruptured membranes, loss of cristae, matrix dissolution, and vacuolization), alterations in mitochondrial membrane potential, and disarray in mitochondrial protein function.

**Figure 2:**
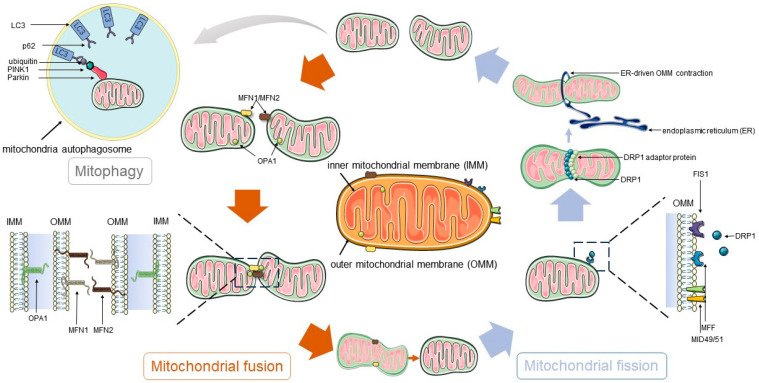
The dynamic balance of mitochondrial fission, fusion, and mitophagy. DRP1 is activated through interaction with its receptors (FIS1, etc.), thereby mediating mitochondrial fission. Mitochondrial fusion is facilitated by MFN1/2 and OPA1. MFN1/2, located in the OMM, mediate the fusion of the outer membrane, while OPA1 is involved in the fusion of the inner membrane, preserving the structure of the cristae and the integrity of the inner membrane.

Mitochondrial fission and fusion are critical processes for maintaining the structural and functional integrity of the mitochondrial network [22] (Figure 2). Dynamin-Related Protein 1 (DRP1), an essential factor in mitochondrial fission, is activated through interaction with its receptors (MFF, MID49, MID51, and FIS1). Changes in the activity of DRP1 and its receptors are closely associated with muscle hypertrophy and atrophy [23]. Mitochondrial fusion is facilitated by Mitofusin 1/2 (MFN1/2) and Optic Atrophy 1 (OPA1). MFN1/2, located in the outer mitochondrial membrane (OMM), mediates the fusion of the outer membrane, while OPA1 is involved in the fusion of the inner membrane, preserving the structure of the cristae and the integrity of the inner membrane. Mitochondrial fusion proteins also significantly impact muscle health. For instance, in mice fed a high-fat diet, there is a significant downregulation of MFN2 and OPA1 in the heart, liver, kidney, spleen, and skeletal muscle, with the most pronounced decrease observed in skeletal muscle [24].

Mitophagy is a selective autophagy mechanism targeting damaged mitochondria, initiated by the recognition of dysfunctional mitochondria. Upon identification and tagging, the key autophagy protein, microtubule-associated protein 1 light chain 3 (LC3), is recruited around the damaged mitochondria to form autophagosomes. These autophagosomes then fuse with lysosomes, leading to the degradation of the impaired mitochondria. Insufficient mitophagy can lead to the accumulation of damaged mitochondria and an increase in reactive oxygen species, causing cellular damage [25]. Conversely, excessive mitophagy may induce the division of healthy mitochondria and the release of proteases from lysosomes, resulting in cell death [26]. Thus, a moderate level of mitophagy is crucial for maintaining mitochondrial function and cell survival. The canonical initiation pathway of mitophagy involves PTEN-induced putative protein kinase 1 (PINK1) and Parkin, key regulatory proteins that participate in the recognition of damaged mitochondria and the initiation of the autophagy process [27] (Figure 2). Parkin is an E3 ubiquitin ligase encoded by the Park2 gene, which can translocate to depolarized mitochondria to initiate mitophagy. PINK1 is localized on the OMM, stimulating the recruitment of Parkin from the cytoplasm, and then removing damaged mitochondria through autophagosomes. Specifically, the activation of Parkin can promote the ubiquitination of many OMM proteins, including MFN1/2, TOM20 (Translocase of the Outer Mitochondrial membrane 20), etc. Subsequently, these ubiquitinated proteins are degraded by the proteasome and autophagy system. Furthermore, mitophagy is closely related to muscle regenerative capacity; a deficiency in PINK1 may affect the differentiation and fate determination of MuSCs, thereby impairing muscle regeneration [28]. In mice, the specific knockout of ATG7 (Autophagy Related 7, a key regulator of autophagy) in muscle leads to severe muscle atrophy, deterioration of neuromuscular junctions, and a shortened lifespan due to abnormal mitophagy [29]. Analysis of individuals with human ATG7 mutations reveals significant neurodevelopmental disorders and poor muscle development [30].

Muscle homeostasis and bone metabolism are intricate and dynamic systems where MQC plays a multifaceted and crucial role. Therapeutic strategies targeting mitochondrial dysfunction, such as the use of antioxidants, modulation of mitochondrial function, and the balancing of gut microbiota, offer new perspectives and potential therapeutic targets for the prevention and treatment of postmenopausal osteoporosis. Nutritional supplementation, exercise training, and pharmacological interventions are effective means of regulating MQC. For instance, omega-3 fatty acids improve muscle health through various mechanisms, including the enhancement of mitochondrial biogenesis and function [31]. High-intensity interval training (HIIT) can ameliorate mitochondrial quality and metabolic function in skeletal muscle by modulating the activity of mitochondrial autophagy and fusion proteins [32]. Medications such as melatonin and atorvastatin exert protective effects on mitochondrial function through antioxidant actions and the regulation of energy metabolism [33,34]. In addition to external interventions, the autocrine effects of myokines also play a role in maintaining the healthy homeostasis of the musculoskeletal system, and this has gradually become known as research into the autocrine effects of skeletal muscle deepens. As a widely studied myokine, we know that both HIIT and melatonin can stimulate the release of irisin, so exploring the role of irisin in maintaining the homeostasis of the musculoskeletal system by maintaining MQC may provide a new perspective on the mechanisms of musculoskeletal diseases [35].

## 3. Myokine Irisin’s Role for Musculoskeletal Disorders

Irisin can promote the proliferation and differentiation of osteoblasts while inhibiting the activity of osteoclasts, which is crucial for the prevention and treatment of bone diseases such as osteoporosis [36,37,38]. Particularly in states of estrogen deficiency, such as in ovariectomized mouse models, an increase in irisin expression is positively correlated with the improvement of trabecular bone density, indicating that irisin may exert a protective role in osteoporosis induced by estrogen deficiency [39]. Moreover, irisin’s regulation of mitochondrial function may also be related to its role in skeletal health. By improving mitochondrial function, irisin may help maintain metabolic balance in bone cells and the stability of bone tissue.

Irisin plays multiple roles in maintaining MQC throughout the entire process. Peroxisome proliferator-activated receptor-gamma coactivator-1 alpha (PGC-1α), a key regulator of mitochondrial biogenesis and ROS metabolism, targets the mitochondrial transcription factor A (TFAM) downstream, which is responsible for controlling the replication and transcription of the mitochondrial genome. Irisin may increase the number of mitochondria in hepatocytes by elevating the expression levels of PGC-1α and TFAM [40]. Furthermore, treatment with irisin has been found to reduce the expression of DRP1 and FIS1 in mouse livers, suggesting that irisin may function by diminishing mitochondrial fission [40]. The research by Liu et al. further confirmed this and found that irisin treatment also upregulates the expression of OPA1 and MFN, indicating that irisin may also promote mitochondrial fusion [41]. Another study revealed that endogenous irisin induced by resistance exercise can activate the PINK1/Parkin-LC3/P62 pathway, regulate mitophagy, and suppress oxidative stress [42]. Thus, irisin can exert an influence at various stages throughout the process of regulating the mitochondrial dynamic balance.

The mechanisms by which irisin regulates MQC pathways are not yet fully understood, but it is known that multiple pathways play a crucial role in maintaining mitochondrial quality homeostasis. For instance, irisin can promote mitochondrial biogenesis and autophagy by activating the AMPK signaling pathway, thereby enhancing mitochondrial quality and function [43,44,45]. Additionally, irisin can upregulate the downstream Akt and ERK1/2 signaling pathways through integrin receptors, which in turn increases mitochondrial biogenesis and morphological repair [46]. Some studies have also demonstrated that irisin activates the PI3K/AKT/mTOR signaling cascade, and inhibition of the PI3K/AKT/mTOR pathway can reverse the effects of irisin on inhibiting mitochondrial fission and promoting fusion [41]. Furthermore, irisin can maintain mitochondrial integrity by affecting the stability and replication of mitochondrial DNA (mtDNA) [47]. For example, in a model of osteoporosis related to diabetes, the restoration of irisin expression helps to alleviate oxidative damage and inhibit pyroptosis, thus protecting skeletal muscle and bone tissue [48]. These findings suggest that irisin may regulate MQC through various pathways and play a significant role in the prevention and treatment of skeletal muscle diseases.

### 3.1. Secretion and Regulation of Irisin

Irisin, a myokine derived from the membrane protein FNDC5, is cleaved and released extracellularly. The precursor of FNDC5 consists of a signal peptide, fibronectin type III domain, a linker region, a hydrophobic transmembrane domain, and a cytoplasmic tail. After processes such as signal peptide cleavage and N-glycosylation, FNDC5 is translocated to the membrane, where it is cleaved to generate irisin [49,50]. Irisin secretion is primarily regulated by the exercise-activated PGC-1α, especially in skeletal muscle [51,52,53]. During the myogenic differentiation process of human muscle cells, irisin secretion increases concurrently with the expression of PGC-1α and myogenin. Additionally, irisin secretion is affected by N-glycosylation, and the blockage of N-glycosylation leading to impaired irisin secretion may be related to the instability of FNDC5 and defects in signal peptide cleavage [54].

Previous research indicates that irisin secretion does not parallel FNDC5 expression. FNDC5 upregulation occurs during the recovery period after exercise, dependent on PGC-1α activation [55], with expression peaking 3 h post-exercise [56]. However, circulating irisin begins to rise during exercise and declines to baseline levels post-exercise [49,57]. These findings suggest that irisin secretion is not related to PGC-1α activation and FNDC5 upregulation. It is hypothesized that irisin secretion may occur through the activation of FNDC5 cleavage rather than the expression of FNDC5 [58,59,60]. However, another study presents an alternative view, identifying a novel secretable FNDC5 (sFNDC5) subtype lacking a transmembrane region in various rat tissues, which exhibits similar or even stronger biological functions compared to irisin, possibly due to its intact structure without cleavage [61], suggesting that uncleaved FNDC5 can also exert relevant biological effects.

As a myokine, irisin functions not only within muscles through autocrine/paracrine mechanisms but is also released into the bloodstream to regulate physiological processes in other tissues as an endocrine factor. For instance, irisin has been shown to promote the transition of white adipose tissue into brown adipose-like cells, a process known as adipose tissue browning. Brown adipose tissue is renowned for its high energy expenditure and thermogenic capacity, thus endowing irisin with potential significance in regulating the energy balance and metabolic health.

Irisin secretion is influenced by various factors, with exercise being the most significant inducer. Studies have shown that irisin levels increase markedly after acute exercise, regardless of age or fitness level [62]. However, the intensity and type of exercise can affect irisin secretion levels. Moderate to high-intensity exercises, including short bouts of high-intensity interval training, can increase circulating irisin levels in healthy individuals and those with different metabolic conditions, depending on the metabolic status and age of the subjects. Acute aerobic exercise more effectively increases irisin levels compared to acute anaerobic exercise, and chronic resistance training has the greatest cumulative effect on irisin levels compared to chronic aerobic and anaerobic training [63]. Secretion of irisin increases only transiently after intense endurance exercise and high-intensity training, peaking immediately post-exercise and gradually returning to baseline levels [64]. In addition, cold exposure has also been found to increase irisin secretion, potentially linked to the activation of brown adipose tissue [53].

Beyond its role as a myokine, irisin is also expressed and secreted in adipose tissue, particularly in subcutaneous and visceral adipose tissues [65]. Irisin secreted by adipose tissue may be involved in the crosstalk between muscle and adipose tissues, mediated by the regulation of FNDC5/irisin expression in both. Additionally, the secretion and function of irisin are modulated by other myokines (such as follistatin or myostatin) and adipokines (such as FGF21 and leptin). This evidence suggests that irisin exists both as a myokine and an adipokine. However, it is noteworthy that subcutaneous and visceral adipose tissues, being secondary production sites for irisin/FNDC5, paradoxically show decreased secretion following long-term exercise training and fasting, which is inconsistent with the regulation of irisin in skeletal muscle [66]. 

### 3.2. Irisin: Sustaining Mitochondrial Homeostasis for Enhanced Musculoskeletal Wellness

#### 3.2.1. The Role of Irisin in Bone/Cartilage Metabolism

The decline in lower limb muscle strength in postmenopausal women is an independent risk factor for increased osteoporosis and fracture risk [67]. A meta-analysis has shown that exercise can significantly improve the vertebral bone density of postmenopausal women and simultaneously enhance their lower limb muscle strength [68]. The causal relationship between lower limb muscle strength and bone density is not limited to postmenopausal women; this correlation has also been scientifically confirmed in a broader population. For instance, in patients with spinal cord injuries, the reduction in bone density is closely related to the denervation of the lower limb muscles [69,70]. Even in adolescents with intact neurological function, short-term plaster fixation of the lower limbs can lead to a rapid decline in total lower limb bone density [71]. These findings underscore the pivotal role of exercise in maintaining the normalization of bone density.

As previously noted, the secretion of irisin increases most significantly after acute aerobic exercise. Consequently, whether it is involved in bone metabolism has become a hot topic of interest for researchers. Several cross-sectional studies on postmenopausal women have shown that decreased serum irisin levels are an independent risk factor for osteoporosis and the risk of fragility fractures [72,73,74]. In addition to clinical studies, animal experiments and in vitro experiments have further confirmed the key role of irisin in bone protection. Mice treated with recombinant irisin have shown a significant increase in cortical bone mass and strength, and irisin stimulates the high expression of FNDC5 in muscle fibers [75]. The expression of FNDC5 in myoblasts of mice that have undergone exercise training increases, and the conditional medium of primary myoblasts from exercised mice can increase the expression of alkaline phosphatase and type I collagen in osteoblasts, which can be reversed by neutralizing antibodies to FNDC5 [76].

Irisin exerts a positive regulatory effect on bone density through various actions on osteoblasts and osteoclasts [77]. Initially, irisin maintains mitochondrial quality by regulating autophagy and mitochondrial biogenesis, showing a particularly significant protective effect on chondrocytes and osteoblasts. Both in vivo and in vitro studies have confirmed that irisin increases the activity of osteoblasts [17,75,77,78]. Estell et al. found that during the differentiation of osteoblast precursors, irisin enhances mitochondrial function, supporting the energy required for their maturation into osteoblasts. Research on bone marrow stromal cells has found that irisin directly activates the transcription factor 4 (ATF4), thereby activating the expression of Runx2 and Sp7, reflecting its direct targeting effect on osteoblasts [75].

Research by Wang et al. has shown that human osteoarthritis articular chondrocytes express reduced levels of FNDC5 and autophagy marker LC3-II, but increased levels of oxidative DNA damage marker 8-hydroxy-2′-deoxyguanosine (8-OHdG) and apoptosis. Irisin can upregulate autophagy and improve the 8-OHdG and apoptosis in damaged cartilage [79]. Besides promoting osteogenesis, irisin also has the ability to inhibit the activity of osteoclasts. Studies have suggested that irisin can promote the proliferation of osteoclast precursor cells but simultaneously inhibit their differentiation into mature osteoclasts [80]. Additionally, research by Zhu et al. has found that irisin significantly reduces the expression of Receptor Activator of Nuclear Factor-κB Ligand (RANKL), thereby suppressing osteoclastogenesis [81]. These findings indicate that irisin may have a dual role in bone metabolism, contributing to the maintenance of bone health.

The pro-osteogenic effect of irisin is particularly significant in states of bone destruction caused by metabolic syndromes such as diabetes, where irisin treatment enhances cell viability and osteogenic effects, mitigating the negative impact of advanced glycation end-products (AGEs) on adipose stem cells. At the same time, irisin regulates mitochondrial function through the SIRT3 pathway, improving oxidative stress in diabetic patients [82].

#### 3.2.2. The Role of Irisin in Brown Adipose Activation

The presence of brown adipose tissue (BAT) in adults and its potential impact on skeletal muscle diseases have been a focus of research in recent years. BAT thermogenesis mainly relies on β-adrenergic-mediated lipolysis activation and subsequent fatty acid degradation through uncoupling protein 1 (UCP1). UCP1 uncouples mitochondrial oxidative phosphorylation to dissipate the electrochemical gradient as heat instead of ATP synthesis, thereby increasing energy expenditure [16,44]. This characteristic leads BAT to play an important role in regulating body temperature and the energy balance. Previous views considered BAT to be present only in infants and disappear rapidly after birth, making it difficult to identify in adults through routine anatomical examination [39,48,83]. However, in recent years, functional BAT has been found in adults, especially in the supraclavicular region, and its activity increases at lower ambient temperatures, decreases after β-adrenergic blocker pretreatment, and is negatively correlated with BMI and total fat [84]. Further research has found that irisin plays a key role in BAT activation and the browning process of white fat. Muscle PGC1-α expression stimulates FNDC5 expression and irisin release. In vitro experiments have found that irisin can stimulate the expression of UCP1 and other brown fat genes in white adipocytes, significantly increasing metabolic capacity and thermogenesis [55]. Thermogenesis is a high-energy-demanding process, which exposes adipocytes to metabolic stress and oxidative damage. The intense activity of mitochondria during thermogenesis makes these organelles highly plastic in terms of metabolic flexibility and structural dynamics. Therefore, maintaining MQC is particularly important for preserving mitochondrial integrity and thermogenic capacity in BAT [85]. Although there is indirect evidence for the role of irisin in reducing oxidative stress and maintaining mitochondrial biofunction in the thermogenic process of BAT and beige fat (browned white fat), further in vivo and in vitro experimental evidence is needed.

#### 3.2.3. The Role of Irisin in Myogenesis

Irisin plays a significant role in promoting muscle growth, enhancing muscle regeneration, activating MuSCs, and potentially regulating metabolism. In vivo experiments have demonstrated that the peripheral application of recombinant irisin in mice increases the expression of FNDC5 in muscle fibers [75]. Moreover, irisin injection can induce skeletal muscle hypertrophy and strengthen the regeneration of skeletal muscle following muscle injury [86]. Irisin administration inhibits MuRF1, Atrogin-1, and myostatin, which are associated with muscle atrophy through the ubiquitin–proteasome system [87]. Further in vitro experiments on mouse myoblasts (C2C12) have shown that irisin significantly upregulates the expression of PGC-1α within myoblasts and increases their mitochondrial content [88], thereby improving myoblast fusion and promoting myogenic differentiation [86]. Irisin also stimulates skeletal muscle hypertrophy by activating the IL-6 signaling pathway, enhancing myogenic differentiation and myoblast fusion, which may be attributed to the activation of MuSCs and increased protein synthesis [86]. Additionally, irisin significantly increases the oxidative metabolism of C2C12 cells, for example, by upregulating metabolic genes PGC-1α, nuclear respiratory factor 1 (NRF1), TFAM, glucose transporter 4 (GLUT4), and mitochondrial uncoupling protein 3 (UCP3), thus promoting mitochondrial biogenesis [89]. Furthermore, irisin enhances the metabolic capacity of skeletal muscle for glucose and lipids by activating the AMPK signaling pathway [53]. Irisin secretion responds to acute exercise in individuals of varying ages and health statuses, but its baseline levels are related to age and health conditions [90]. These findings suggest that irisin plays a role not only in the post-exercise remodeling of skeletal muscle but may also serve as a potential therapeutic target for muscle atrophy and metabolic diseases.

As a factor released by skeletal muscle following exercise, irisin has effects not only through endocrine actions on bones, fat, and distant organs but also through its autocrine/paracrine actions, which have garnered considerable interest. Interestingly, the primary site of irisin expression may not be the muscle cytoplasm but the nerve sheath that innervates skeletal muscle [88,91]. Additionally, other highly metabolically active tissues, such as the liver, also express irisin. This evidence further supports the notion that the effects of irisin may be mediated through autocrine/paracrine mechanisms for metabolic regulation.

**Figure 3:**
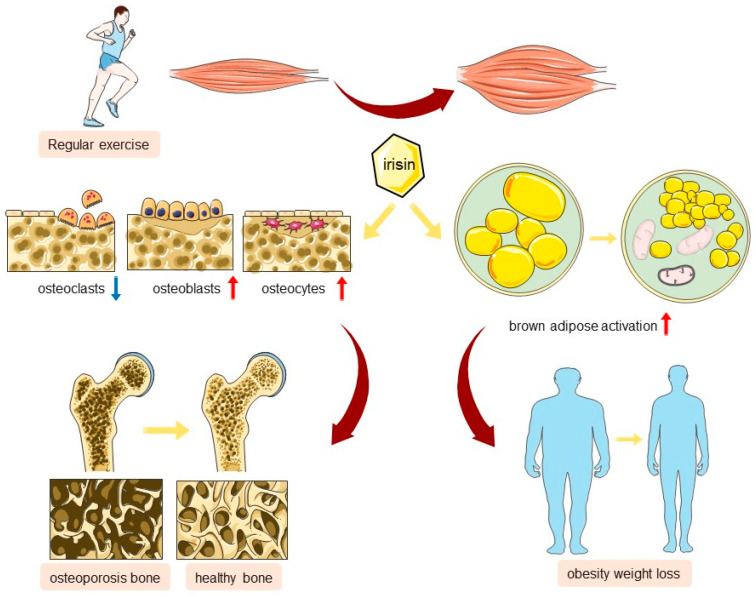
The regulation of MQC by irisin and its role in musculoskeletal diseases. Irisin plays a crucial role in promoting musculoskeletal health by maintaining MQC, including mitigating obesity, and slows the progression of diseases like osteoporosis by enhancing osteoblasts, inhibiting osteoclasts, and activating brown adipose cells.

In conclusion, the myokine irisin, secreted as a result of physical activity, is pivotal in fostering musculoskeletal health by preserving MQC. It achieves this by a variety of mechanisms, such as bolstering the function of osteoblasts, curbing the activity of osteoclasts, and stimulating brown adipose tissue. These actions contribute to the mitigation of obesity and the amelioration of conditions like osteoporosis (Figure 3). With further research, irisin may become a new strategy adopted for the treatment of chronic diseases in the musculoskeletal system in the future.

### 3.3. Irisin Plays a Role in Maintaining Mitochondrial Homeostasis in Musculoskeletal Diseases

#### Mitochondrial Dysfunction in Adolescent Idiopathic Scoliosis

Adolescent idiopathic scoliosis (AIS) is a common spinal deformity. The paraspinal muscles play a critical role in spinal stability. Alterations in these muscles manifest as reduced muscle spindle density, asymmetric electromyographic activity, and shifts in muscle fiber types, leading to poor endurance and increased fatigue in AIS patients. Recent research has increasingly focused on physiological changes in the paraspinal muscles, such as fiber alterations, myogenesis defects, and electrophysiological abnormalities. These alterations may be interconnected, with oxidative stress potentially playing a role in exacerbating these conditions, leading to apoptosis of muscle cells and disruptions in the regulation of muscle development [92].

A study has found an association between Lon Peptidase 1 (LONP1) and the incidence of AIS [93]. LONP1 encodes a mitochondrial matrix protein [94]. In conditions of muscle inactivity or disuse, the activity of LONP1 declines, mitochondrial autophagy is impaired, and this leads to mitochondrial dysfunction and a reduction in muscle mass [95]. Similar reports exist for congenital scoliosis, with genetic studies finding a significant reduction in circulating cell-free mtDNA in patients with congenital scoliosis [96]. A case report also described a severe syndrome of congenital scoliosis with a homozygous mutation in the TBXT gene associated with a novel heteroplasmic mitochondrial mutation in the MT-ND3 (Mitochondrially Encoded NADH: Ubiquinone Oxidoreductase Core Subunit 3) gene. The authors propose that mtDNA mutations may have a potential role in the severe phenotype of congenital scoliosis [97]. In summary, as a disease with unknown etiology that progresses rapidly during the growth period, research on AIS has increasingly focused on muscle asymmetry. Undoubtedly, the quality control of mitochondria in skeletal muscle is an important part of maintaining the homeostasis of paraspinal muscles and may be the key to unraveling the mechanisms of this disease.

Melatonin is an antioxidant that targets mitochondria. It accumulates in high concentrations within the mitochondria and helps maintain mitochondrial membrane potential and protect mitochondrial function by scavenging ROS, inhibiting the mitochondrial permeability transition pore (MPTP), and activating uncoupling proteins (UCPs). Additionally, melatonin regulates mitochondrial dynamics and maintains MQC. For instance, melatonin can reduce mitochondrial fission and enhance fusion to promote mitochondrial biogenesis, while also enhancing mitophagy to maintain the dynamic balance of mitochondria [98]. The association between abnormal melatonin signaling pathways and the progression of AIS has been studied at the genetic level. The melatonin receptor MT2 is expressed at lower levels in bone marrow mesenchymal stem cells of patients with AIS, leading to abnormal cellular responses to melatonin during the processes of osteogenesis and chondrogenesis, which may be related to abnormal membrane and endochondral ossification, as well as skeletal growth [99].

Another study on bone marrow mesenchymal stem cells from AIS patients supports this conclusion and identifies a key gene, SPRY4, which is significantly under-expressed in AIS bone marrow mesenchymal stem cells. This under-expression significantly downregulating the mitogen-activated protein kinase (MAPK) pathway. Consequently, melatonin, which normally promotes osteogenic differentiation, may instead result in impaired osteogenic differentiation of mesenchymal stem cells (MSCs) in AIS [100].

Drawing from a multitude of clinical studies and animal experiments, the association between melatonin and myokines, including irisin, may constitute one of the mechanisms of its action. The protective effect of melatonin in musculoskeletal diseases is partially mediated by irisin. In a retrospective study, a correlation was observed between several myokines related to exercise and muscle mass, including irisin, and the failure of brace treatment in patients with AIS [101]. Rats treated with melatonin had a significant increase in serum irisin levels, along with weight loss, decreased serum cholesterol, and increased brown fat, resembling the changes seen in rats treated with irisin [35]. Another study reported that after the normal function of the angiotensin II–melatonin axis was disrupted in rats using arsenic trioxide, there was atrophy of the gastrocnemius muscle, with a significant decrease in the myokines insulin-like growth factor-1 (IGF-1) and irisin, and a notable increase in myostatin, indicating a significant correlation [102]. However, research directly linking myokines such as irisin to the pathogenesis of scoliosis is currently lacking. As a key factor in the regulation of mitochondrial quality, irisin may play a pivotal role in the initiation and progression of these musculoskeletal diseases, warranting further investigation.

Osteoporosis is a systemic skeletal disease characterized by reduced bone mass and deterioration of bone microstructure, leading to increased fragility and a heightened risk of fractures. A substantial body of evidence from clinical studies supports the positive role of irisin in maintaining bone mass (Table 1). Exposure to prednisone during pregnancy reduces FNDC5 expression in fetal skeletal muscle, simultaneously increasing its susceptibility to osteoporosis [14]. In another study, the authors suppressed the expression of FNDC5 by overexpressing a microRNA (miR-150-5p), resulting in the inhibition of the p38/MAPK pathway and impairment of osteogenic differentiation in mice. The subsequent overexpression of FNDC5 reversed the inhibition of this pathway, thereby demonstrating the crucial role of FNDC5 in the signaling cascade of osteogenic differentiation [103].

Mitochondrial dysfunction is implicated in the pathogenesis of postmenopausal osteoporosis. A study has identified a decrease in circulating mtDNA as an independent risk factor for reduced bone mineral density in the femoral neck of postmenopausal women, highlighting the clear role of mitochondrial dysfunction in the pathophysiological mechanisms of osteoporosis in postmenopausal women [109]. As research advances, various molecular biomarkers related to mitochondrial function, such as AMP-activated protein kinase (AMPK), forkhead box transcription factor O3 (FOXO3), NDUFA4 mitochondrial complex associated like 2, adenosine 2a receptor, and plant homeodomain finger protein 23, have been identified as closely associated with the development of osteoporosis [110]. Mitochondrial dysfunction affects the balance between bone formation and resorption by impacting processes such as mitochondrial fusion and fission, biogenesis, and autophagy, playing a significant role in the disease’s mechanism [7].

Postmenopausal estrogen deficiency leads to chronic oxidative-stress-mediated senescence of bone marrow mesenchymal stem cells (BMMSCs), considered a significant hallmark of osteoporosis onset, where MQC plays a key role [111]. Studies suggest that genistein from soy isoflavones can alleviate the senescence of BMMSCs in ovariectomized rats by mediating ERRα-dependent mitochondrial biogenesis and autophagy [111]. MitoTEMPO, a mitochondria-targeted antioxidant, can ameliorate the senescence of BMMSCs in ovariectomized rats, indicating that the restoration of mitochondrial function and quality control are key determinants in regulating oxidative stress and cellular senescence [112]. Furthermore, evidence suggesting that bone loss due to estrogen deficiency is caused by changes in mitochondrial activity. 17-beta-estradiol (E2) influences early metabolic changes in osteoclast precursor cells by affecting mitochondrial metabolism, potentially related to mitochondria-mediated apoptotic pathways [113]. RANKL, a key cytokine for osteoclast differentiation, binds to ECSIT in the Toll pathway of osteoclast precursor cells, recruiting the adapter protein TRAF6 and activating the signaling pathway that stimulates mitochondria and upregulates osteoclast activity [114]. E2 prevents the effects of RANKL on mitochondria and promotes mitochondria-mediated apoptotic cell death.

Lastly, other evidence suggests that mitochondrial dysfunction is involved in the pathogenesis of postmenopausal osteoporosis. For instance, postmenopausal iron metabolism disorders, particularly iron overload, can lead to bone loss by affecting mitochondrial function [115]. Icariin, as an antioxidant, protects bone cells from iron-overload-induced oxidative stress, inhibiting bone loss, with its mechanism involving the preservation of mitochondrial membrane potential and the reduction of ROS [116]. A wealth of research indicates that irisin plays a pivotal protective role in various diseases such as myocardial infarction, heart failure, lung ischemia–reperfusion, and Parkinson’s disease by ameliorating mitochondrial dysfunction, inhibiting oxidative stress, and metabolic imbalance [41,46,117,118]. Current research on irisin in musculoskeletal diseases is mostly limited to the regulation of osteogenic/osteoclastic genes and pathways, such as the ERK, p38, and AMPK signaling pathways [17,77], which are largely intertwined with MQC. Therefore, we have reason to speculate that the positive role of irisin in MQC is the main mechanism of its anti-osteoporotic effect. However, this argument still requires further confirmation through more in vivo and in vitro studies (Figure 3).

## 4. Debates on Irisin’s Role and Measurement in Humans

Since Boström and colleagues first reported irisin in 2012, a myokine induced by exercise, debates surrounding its physiological role have been ongoing [55]. Irisin is considered a highly conserved protein, with the FNDC5 gene from species such as rats, mice, chimpanzees, gorillas, and orangutans showing a conserved ATG translation start codon, except for the human sequence. In humans, the start codon is ATA, encoding isoleucine (I) instead of the conserved ATG that encodes methionine (M) [119]. Raschke has suggested that the unique ATA start codon in humans results in a significantly lower translation efficiency of the FNDC5 transcript compared to species with ATG at this position [120]. Additionally, Raschke’s incubation of human adipocyte precursors with recombinant FNDC5 and irisin revealed no change in mRNA expression of key proteins for adipose tissue browning. These findings raise the question of whether conclusions from animal and cellular experiments on irisin can be extrapolated to humans, which requires cautious consideration.

Measurements of irisin levels in humans have also been questioned. A gene chip probe analysis by Timmons et al. on a large cohort (200 subjects) found that while the majority benefited metabolically from an exercise training program, an increase in muscle FNDC5 was only observed in older subjects and was not related to metabolic status, nor was there evidence to support irisin stimulating UCP1 expression [121].

Furthermore, early detection of irisin relied on Western blotting for deglycosylated FNDC5, but inaccurate antibodies led to incorrect identification of irisin protein bands [55]. The subsequent discontinuation of these antibodies and the introduction of new ones have continued to be met with skepticism from researchers. For the detection of circulating irisin, Western blot experiments have been unable to reproduce results, and there is methodological confusion and challenge. According to current literature reports, it can be concluded that there is currently no reliable method for detecting circulating irisin. Moreover, the reliability of ELISA kits for irisin/FNDC5 has also been questioned, with published human serum irisin studies showing levels ranging from 0.01 to 2000 ng/mL, indicating a significant discrepancy between studies [122]. Similarly, the accuracy of ELISA kits for mice is not optimistic. For example, compared to the more precise quantitative mass spectrometry method, ELISA determined that the circulating irisin levels in the same group of mice were more than 2500 times higher [59].

## 5. Summary and Prospects

Mitochondria, as the central hub of energy metabolism, maintain a high degree of dynamism and stable quantity and quality control, playing a crucial role in the timely clearance of damaged mitochondria and the replenishment through biogenesis. This maintenance of mitochondrial homeostasis is intimately linked to cellular activity, especially in energy-metabolism-rich tissues such as skeletal muscle and brown adipose tissue. Irisin, as a myokine secreted by skeletal muscle, enhances the energy metabolism capacity of muscle cells by promoting mitochondrial biogenesis and function, thereby improving muscle strength and endurance. Additionally, irisin protects skeletal muscle cells from oxidative stress damage by regulating mitochondrial autophagy, maintaining muscle health, and preventing muscle atrophy. MQC plays a crucial role in maintaining muscle health, and its dysregulation is associated with a variety of diseases. Irisin affects the dynamic balance and autophagy of mitochondria, which has an important role in the health of the musculoskeletal system and the prevention of diseases.

However, it is essential to recognize the limitations of current research on irisin and question the extrapolation of research findings to humans. What drives the cleavage of FNDC5, through what mechanisms does exercise promote increased levels of irisin, and can secretory FNDC5 be secreted intact without cleavage to exert stronger physiological effects? These unresolved questions will dictate future research directions for irisin. There is also an urgent need to significantly improve detection methods for FNDC5/irisin.

In summary, as research progresses, the pivotal role of mitochondrial homeostasis in numerous diseases and the aging process continues to be revealed. Irisin, as an endogenous myokine, holds promise as a potential solution for addressing issues related to poor mitochondrial quality control.

## Figures and Tables

**Table 1:** Clinical studies on the role of irisin in regulating bone mass.

Date of Publish	Research Type	Sample Size	Subjects	Conclusion	Reference
24 January	cross-section study	103	Children and adolescents	Serum irisin levels are associated with bone quality in children and adolescents.	[104]
23 February	cross-section study	138	Postmenopausal women	Serum irisin levels are positively correlated with BMD. Low serum irisin levels are associated with a high risk of falls and low muscle strength.	[105]
23 July	cross-section study	74	Patients with chronic kidney disease	Serum irisin levels are positively correlated with femoral BMD.	[106]
21 December	cross-section and case–control study	430 (215:215)	Postmenopausal women with/without hip fractures	Decreased circulating irisin serum levels are associated with an increased risk of hip fractures related to osteoporosis.	[72]
21 February	cross-section study	62	Elderly patients with arthroplasty surgery	Circulating irisin levels are negatively correlated with the incidence of age-related osteoporosis.	[107]
15 April	cross-section study	72 (36:36)	Overweight patients with/without osteoporosis	Circulating irisin levels are negatively correlated with vertebral fragility fractures.	[108]
14 May	matched case–control study	125 (50:25; 25:25)	Postmenopausal women with low bone mass and osteoporosis	Circulating irisin levels are negatively correlated with osteoporotic fractures.	[74]
